# Test your knowledge and understanding

**Published:** 2018-02-08

**This page is designed to help you to test your own understanding of the concepts covered in this issue, and to reflect on what you have learnt.**

**Figure:**
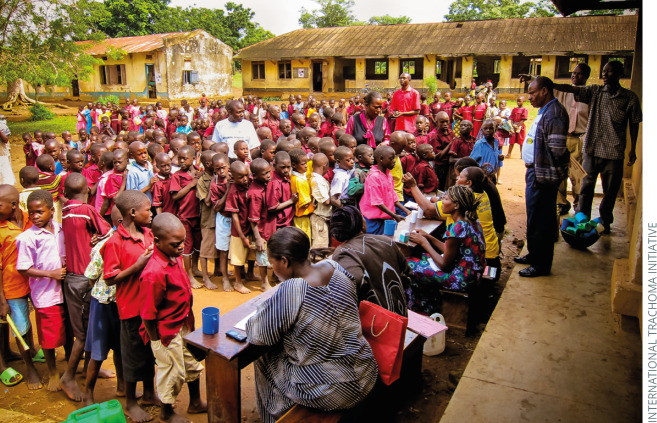
School children line up to receive Zithromax. UGANDA

We hope that you will also discuss the questions with your colleagues and other members of the eye care team, perhaps in a journal club. To complete the activities online – and get instant feedback – please visit **www.cehjournal.org**

**Tick ALL that are TRUE**

**Question 1 Visual Impairment**
  □ **a** Visual impairment is defined as <6/18 to 6/60 in the better eye at presentation.
  □ **b** The major cause of visual impairment in the world is uncorrected refractive error.
  □ **c** In 2015 there were an estimated 253 million visually impaired people in the world.
  □ **d** There are more visually impaired men than women, approximately 55% to 45%.
  □ **e** The prevalence of vision impairment increased in the world from 1990 to 2015.
**Question 2 Cataract Indicators**
  □ **a** Cataract Surgical Coverage (CSC) is a measure of the quality of a cataract operation.
  □ **b** The main risk factor for incidence of visually impairing cataract is age.
  □ **c** Cataract Surgical Rate (CSR) depends mainly on the number of ophthalmologists per million population.
  □ **d** A poor outcome after cataract surgery (defined as <6/60 vision at presentation 6 weeks after surgery) is nearly always due to surgical complications.
  □ **e** The useful indicators in monitoring a cataract service are cataract surgical rate, cataract surgical coverage and cataract outcome.
**Question 3 Childhood eye disease**
  □ **a** Vitamin A deficiency is no longer a public health problem in the world.
  □ **b** Retinopathy of prematurity occurs only in high-income countries.
  □ **c** Rubella is a preventable cause of congenital cataract.
  □ **d** Uncorrected hypermetropia is the commonest cause of visual acuity <6/12 in school children.
  □ **e** Low vision services and aids are often needed after surgery for cataract in children.
**Question 4 Infectious eye diseases**
  □ **a** The treatment for onchocerciasis is Mectizan (ivermectin) given annually to communities where onchocerciasis is endemic.
  □ **b** Trachoma is caused by *Chlamydia trachomatis* serotypes A, B and C.
  □ **c** In communities where the prevalence of TF in children aged 1–9 years is 15%, azithromycin treatment to the community is recommended annually for 3 years.
  □ **d** Trichiasis surgery for TT can be performed in health clinics by eye assistants trained in TT surgery.
  □ **e** The number of blind people from both onchocerciasis and trachoma has decreased between 1990 and 2015.

## ANSWERS

1. b and c are TRUE. Visual impairment is defined as <6/18 to no light perception in the better eye at presentation; it includes moderate and severe VI and blindness. It is estimated that 55% of all visually impaired people in the world are women. The prevalence of VI is estimated to have decreased from 4.58% to 3.38% between 1990 and 2015.
2. b and e are TRUE. CSC is a measure of the coverage, that is quantity of cataract services. The CSR depends on a number of factors including barriers to access e.g. cost, distance; and the indication for surgery. If there are insufficient ophthalmologists then the CSR may be low, however the number of ophthalmologists is not the MAIN factor. Some countries in Latin America and Eastern Europe have many ophthalmologists but relatively low CSR. Poor outcome can be due to uncorrected refractive error; co-existing eye pathology e.g. glaucoma; or surgical complications. It is not true to say poor outcome is “nearly always due to surgical complications”.
3. c and e are TRUE. Vitamin A deficiency remains a public health problem in some countries and is a preventable cause of childhood mortality and visual loss. ROP is seen in Latin America, Eastern Europe and urban centres of middle income countries in Asia and Africa being associated with increased survival of low birth weight babies in neonatal units. Uncorrected MYOPIA is the major cause of decreased acuity in school children.
4. All are TRUE.

